# Highly polarized photoluminescence from c-plane InGaN/GaN multiple quantum wells on stripe-shaped cavity-engineered sapphire substrate

**DOI:** 10.1038/s41598-019-44519-2

**Published:** 2019-06-04

**Authors:** Jongmyeong Kim, Seungmin Lee, Jehong Oh, Jungel Ryu, Yongjo Park, Seoung-Hwan Park, Euijoon Yoon

**Affiliations:** 1 Department of Materials Science and Engineering, Seoul National University, Seoul, 08826 Korea; 2 Department of Electronics Engineering, Catholic University of Daegu, Gyeongbuk, 38430 Korea; 3 Research Institute of Advanced Materials, Seoul National University, Seoul, 08826 Korea; 4 Inter-university Semiconductor Research Center, Seoul National University, Seoul, 08826 Korea

**Keywords:** Applied physics, Inorganic LEDs

## Abstract

Highly polarized photoluminescence (PL) from c-plane InGaN/GaN multiple quantum wells (MQWs) grown on stripe-shaped cavity-engineered sapphire substrate (SCES) was realized. The polarization ratio was as high as 0.74 at room temperature. High-resolution X-ray reciprocal space mapping measurements revealed that the InGaN quantum wells on GaN/SCES template were under considerable anisotropic in-plane strain states of −1.178% and −1.921% along the directions perpendicular and parallel to the stripe-pattern, respectively. The anisotropic strain states were attributed to the anisotropic alignment of cavity-incorporated sapphire nano-membranes, which accommodated both anisotropic elastic relaxation in the InGaN quantum well plane as well as the graded elastic relaxation along the vertical direction in the GaN template adjacent to the InGaN/GaN MQWs. The partial strain relaxation in the InGaN wells also contributed to reduction of quantum confined Stark effect, resulting in four times higher PL intensity than InGaN/GaN MQWs on planar sapphire substrate. From theoretical calculations based on *k∙p* perturbation theory, it was found that fundamental origin of the polarized optical emission was strain-induced modification of valence band structures of the InGaN/GaN MQWs on the SCES. This study will allow us to realize light emitting diodes with highly polarized emission with conventional c-plane sapphire substrates by strain-induced valence band modification.

## Introduction

GaN-based light emitting diodes (LEDs) have been widely used in many lighting applications due to their high efficiency and substantial energy savings. LEDs also have many advantages such as control of color temperature, pulsed operation, dimming, far-field emission pattern, and linearly polarized emission, which are not possible in traditional lighting sources such as incandescent and fluorescent lamps^1^. However, the phosphor-converted white light from GaN-based LEDs produce unpolarized light. As a result, in present, for the liquid crystal displays (LCDs) application, unpolarized light source and the additional absorbing front polarizers are simultaneously required, which results in significant losses of light and compactness^2^. Therefore, if it is possible to generate the linearly polarized light emission with high efficiency, the efficiency and compactness of LCDs would be greatly improved.

The linearly polarized light emissions have been observed from non- or semi-polar InGaN/GaN single quantum well and multiple quantum wells (MQWs) grown on non- or semi-polar bulk GaN substrates due to the valence band modification induced by inherent anisotropic in-plane strain in non- or semi-polar InGaN/GaN MQWs^3–17^. However, limited size and high cost of the bulk GaN substrates act as barriers to mass production of LEDs with polarized emission. Furthermore, although the polarized light emissions have been also observed in MQWs with non- or semi-polar hetero-epitaxial structures^17,18^, the structures are highly defective. Thus, it will be very important to realize linearly polarized light emission with the commonly used c-plane InGaN/GaN MQWs on c-plane sapphire substrate to resolve these problems.

However, the c-plane InGaN/GaN MQWs exhibit no polarized light emission due to the isotropic in-plane symmetry and resultant isotropic in-plane strain^19^. To obtain the polarized light emission from the c-plane InGaN/GaN MQWs, some technical endeavors have been reported^20–24^. Zhuang *et al*. proposed a top-down fabrication of asymmetric nanostructures to induce anisotropic in-plane strain in c-plane InGaN/GaN MQWs^20^. However, an additional plasma etching process could cause plasma damage and the reduction of active area. Other methods such as packaging for side wall emission^21,22^ or the integration of metallic nano-gratings have been investigated^23,24^. However, these methods also required complex post-processes for extracting the polarized light emission or resulted in the loss of light due to reflection at the interface between LEDs and the metallic nano-gratings.

The c-plane InGaN/GaN MQWs suffer from quantum confined Stark effect (QCSE) due to lattice mismatch strain-induced piezoelectric fields, which leads to reduced overlap of electron and hole wave functions and resultant reduction of internal quantum efficiency^25,26^. Although strain relaxation in InGaN wells and resultant improvement in optical properties of c-plane InGaN/GaN MQWs have been reported, the structures have been limited to top-down etched nano-structures accompanied by plasma damage and reduction of active area^27–30^.

It has been theoretically and experimentally reported that c-plane GaN-based semiconductors under anisotropic in-plane strain (uniaxial strain perpendicular to c-axis) exhibited modified valence band structures and resultant optical properties^31–33^. Therefore, if it is possible to simply render and control the in-plane strain states in continuous c-plane InGaN/GaN MQWs grown on c-plane sapphire substrate, the linearly polarized emission with high efficiency could be realized at an affordable production cost. Recently, we demonstrated the anisotropic in-plane strain and resultant polarized photoluminescence (PL) from a continuous c-plane GaN layer grown on stripe-shaped cavity-engineered sapphire substrate (SCES)^34^.

In this study, we report a realization of the polarized light emission from c-plane InGaN/GaN MQWs grown on the GaN/SCES template. The polarization ratio as high as 0.74 was obtained. The fundamental origin of polarized light emission was investigated by high-resolution X-ray reciprocal space mapping (RSM) and theoretical calculations based on *k∙p* perturbation theory. This study gives opportunities to realize LEDs with highly polarized emission from the c-plane InGaN/GaN MQWs by strain-induced modification of valence band structures in III-nitride semiconductors.

## Results and Discussion

**Figure 1:**
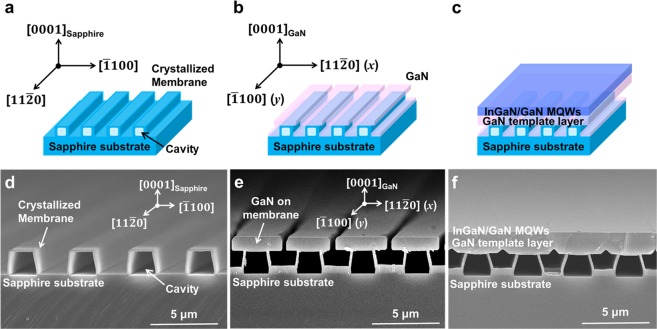
Schematic diagrams and SEM images of SCES and epilayer growth on the SCES. (**a**) Schematic diagram of SCES. (**b**) Schematic diagram of GaN grown on SCES after grown time of 60 min. (**c**) Schematic diagram of InGaN/GaN MQWs grown on the continuous GaN/SCES template. Corresponding SEM images of (**d**) SCES and (**e**) GaN grown on SCES after grown time of 60 min, and (**f**) InGaN/GaN MQWs grown on the continuous GaN/SCES template.

Figure 1 shows schematic diagrams and corresponding scanning electron microscope (SEM) images of SCES, c-plane GaN grown on the SCES, and InGaN/GaN MQWs grown on the GaN/SCES template, respectively. Stripe-shaped cavity-incorporated sapphire nano-membranes were orderly arranged on a planar sapphire substrate along the [11$\bar{2}$0] direction of sapphire as shown in Fig. 1a,d. Figure 1b,e show laterally grown stripe-shaped GaN array on each membrane due to the anisotropic growth behavior of GaN after the 60 min growth in a metal-organic chemical vapor deposition (MOCVD) chamber. The directions perpendicular and parallel to the stripe-shaped pattern corresponded to [11$\bar{2}$0] (*x*) and [$\bar{1}$100] (*y*) directions of GaN, respectively, as shown in Fig. 1b,e. During the additional 30 min growth, the GaN on each membrane was merged with adjacent ones, resulting in the continuous GaN/SCES template. The fabrication process of SCES and growth of continuous GaN template layer on the SCES were described in Method section and elsewhere^34^. Five-period InGaN/GaN MQWs were subsequently grown on the continuous GaN/SCES template as shown in Fig. 1c,f. The orderly arranged stripe-shaped cavity-incorporated nano-membrane structures between epitaxial layers and sapphire substrate were well maintained after the growth of the InGaN/GaN MQWs. For comparison, the InGaN/GaN MQWs were also grown on a GaN/planar sapphire substrate template as a reference sample in the same batch.

**Figure 2:**
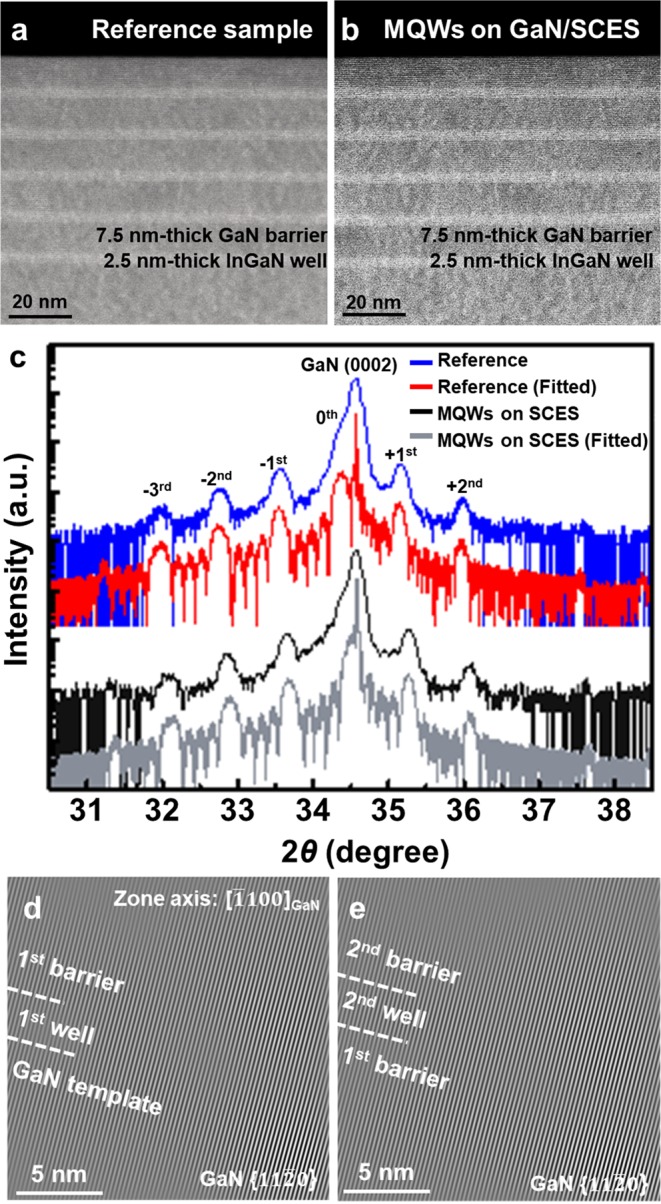
Structural properties of InGaN/GaN MQWs on GaN/SCES template and reference sample. Cross-section STEM images of (**a**) 5-period InGaN/GaN MQWs of reference sample and (**b**) on SCES (**c**) ω-2θ scan profiles of InGaN/GaN MQWs on GaN/SCES template and reference sample, and the corresponding fitted profiles. Reconstructed TEM images at the interfaces of (**d**) 1^st^ GaN barrier/1^st^ InGaN well/GaN template and (**e**) 2^nd^ GaN barrier/2^nd^ InGaN well/1^st^ GaN barrier of InGaN/GaN MQWs on GaN/SCES template.

Structural properties of the reference sample and InGaN/GaN MQWs grown on the GaN/SCES template were analyzed by scanning transmission electron microscopy (STEM), high-resolution X-ray diffraction (XRD) and X’pert Epitaxy Smoothfit software^35^, and high-resolution TEM. Figure 2a,b show STEM images of the reference sample and five-period InGaN/GaN MQWs grown on the GaN/SCES template, respectively. Sharp interfaces between InGaN well and GaN barrier layers were observed as shown in Fig. 2a,b. For both samples, thicknesses of InGaN well and GaN barrier layers were measured to be 2.5 nm and 7.5 nm, respectively. Figure 2c shows ω-2θ scan profiles of the samples measured by high-resolution XRD measurements and corresponding curve fit. For both samples, GaN template layer peaks and InGaN/GaN MQWs satellite peaks were clearly and sharply defined, indicating successful growth of InGaN/GaN MQWs and sharp interfaces between epitaxial layers. For the InGaN/GaN MQWs on the GaN/SCES template, it was worth noting that InGaN/GaN MQWs satellite peaks were slightly shifted compared to those of the reference sample. To investigate indium compositions and extent of lattice mismatch strain relaxation in InGaN/GaN MQWs of the samples, fitting process was conducted. For the fitting process, we assumed that lattice mismatch strain in GaN template layer on planar sapphire substrate was fully relaxed after the growth of the 1.7 μm-thick GaN template layer. For the InGaN/GaN MQWs on the GaN/SCES template, it was assumed that InGaN/GaN MQWs were grown on fully relaxed GaN template. Reflecting the thicknesses of InGaN well and GaN barrier layers measured by STEM, indium compositions were estimated by 19.5% for the both samples. Partial relaxations of lattice mismatch strain of 44% and 12% along the *x-* and *y*-directions, respectively, were observed only in InGaN wells grown on the GaN/SCES template. High-resolution TEM analysis was conducted to investigate the origin of the partial strain relaxation in the InGaN wells grown on the GaN/SCES template. We investigated whether misfit dislocations were generated at the interface between the InGaN/GaN MQWs and GaN template layer as well as in the InGaN/GaN MQWs. Figure 2d,e show reconstructed TEM images by selected Fourier filtering near the interfaces of 1^st^ GaN barrier/1^st^ InGaN well/GaN template layer and 2^nd^ GaN barrier/2^nd^ InGaN well/1^st^ GaN barrier, respectively. Considering the lattice mismatches of about 2% between In_*x*_Ga_1−*x*_N (*x* = 19.5%) and GaN, misfit dislocations should be generated at every 50 interplanar spacing on average. However, misfit dislocations were not observed at both regions over 80 interplanar spacing, indicating the pseudomorphic growth of InGaN/GaN MQWs on the GaN template layer, as shown in Fig. 2d,e.

**Figure 3:**
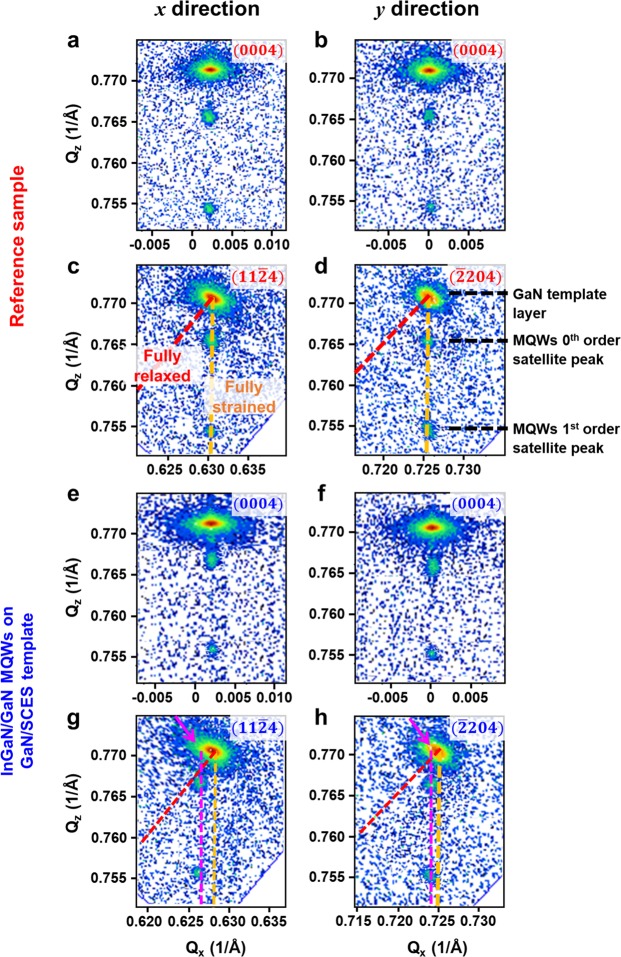
Reciprocal space maps of InGaN/GaN MQWs on GaN/SCES template and reference samples. Reciprocal space maps of reference sample around (0004) reflections (**a**) along *x* direction and (**b**) along *y* direction, and asymmetric reflections around (**c**) (11$\bar{2}$4) and (**d**) ($\bar{2}$204). Reciprocal space maps of InGaN/GaN MQWs and GaN template layer grown on SCES around (0004) reflections (**e**) along *x* direction and (**f**) along *y* direction, and asymmetric reflections around (**g**) (11$\bar{2}$4) and (**h**) ($\bar{2}$204).

To further investigate the structural properties of epitaxial layers of the samples, high-resolution X-ray RSM measurements were conducted. Figure 3a,b show symmetric (0004) reflections from of the reference sample along the *x-* and *y*-directions, respectively. Those from the InGaN/GaN MQWs on the GaN/SCES template were shown in Fig. 3e,f, respectively. Symmetric reflections provide the information related to the crystallographic tilt induced by miscut of substrate or by dislocations^36^. Notable crystallographic tilt angle (*α*) of 0.17°, $\alpha ={\tan }^{-1}({Q}_{x}/{Q}_{z})$, were observed only along the *x*-direction of the both samples as shown in Fig. 3a,e. The crystallographic tilt was comparable with the miscut angle (toward m-axis) of sapphire substrate of 0.2° and consistent with the value reported in our previous study^34^. Figure 3c,d show asymmetric (11$\bar{2}$4) and ($\bar{2}$204) reflections from the reference sample, respectively. Figure 3g,h show those from the InGaN/GaN MQWs on the GaN/SCES template, respectively. From the RSM theory, strain states in MQWs can be accurately analyzed by relative positions of reciprocal lattice points (RLPs) of MQW satellite peaks with respect to those of underlying layers^36^. For example, the RLPs of the InGaN/GaN MQW satellite peaks on dashed red or orange lines in asymmetric reflections such as in Fig. 3c indicate that the corresponding layers are under fully-relaxed or fully-strained states with respect to the GaN template layer, respectively. RLPs of partially relaxed layers appear between the red and orange lines. Notable differences in shapes of RLPs were observed from the RSM measurements. For the reference sample, the 0^th^ and the 1^st^ satellite peaks of InGaN/GaN MQWs lied on the dashed orange line, which means that in-plane interplanar spacing of InGaN/GaN MQWs was the same with that of the GaN template layer as shown in the Fig. 3c,d. In other words, InGaN/GaN MQWs were pseudomorphically grown on the underlying GaN template layer on the planar sapphire substrate, and they were under fully-strained states. On the other hand, the RLPs of InGaN/GaN MQWs satellite peaks were slightly shifted from that of the GaN template layer, as shown in the Fig. 3g,h with dashed magenta lines. In addition, the RLPs of the GaN template layer were somewhat elongated toward lower *Q*_*x*_ and higher *Q*_*z*_ as shown with magenta arrows, implying locally inhomogeneous strain states in the GaN template layer. However, it was worth noting that the *Q*_*x*_ coordinates of the MQWs satellite peaks were the same with that of the elongated RLPs of the GaN template layer, which indicates that the InGaN/GaN MQWs were pseudomorphically grown on the locally deformed GaN template layer. The in-plane interplanar spacing (*d*) and corresponding strains in InGaN wells along the *x-* and *y*-directions (*ε*_*xx*_ and *ε*_*yy*_) were calculated by reflecting the tilt correction (See Supplementary Information)^37^. As summarized in Table 1, strains along *x* and *y* directions in the InGaN wells of the reference sample were found to be −2.197% and −2.197%, respectively, indicating the isotropic in-plane strain states due to its in-plane symmetry as expected. On the other hand, the InGaN wells grown on the GaN/SCES template were under significant anisotropic in-plane strains of −1.178% and −1.921% along the *x* and *y* directions, respectively. Strain relaxation (*R*) [defined as $({d}^{InGaN}-{d}^{GaN})/({d}_{bulk}^{InGaN}-{d}_{bulk}^{GaN})$]^38^ in InGaN wells on the SCES were found to be 43.3% and 12.5% along *x-* and *y*-directions, where *d*^*InGaN*^ and *d*^*GaN*^ are interplanar spacing of InGaN and GaN, respectively. The results were comparable with the values obtained from the high-resolution XRD measurements, and means considerable anisotropic in-plane strain states in InGaN wells grown on the GaN/SCES template (See Supplementary Information).

**Table 1:** Interplanar spacing (*d*) and corresponding strains along the *x* and *y* directions (*ε*_*xx*_ and *ε*_*yy*_) of the InGaN wells of the samples.

Directions	Reference sample				InGaN wells on GaN/SCES template			
	${{\boldsymbol{d}}}_{(11\bar{{\bf{2}}}0)}$(Å)	${{\boldsymbol{d}}}_{(\bar{{\bf{2}}}200)}$(Å)	*ε*_*xx*_ (%)	*ε*_*yy*_ (%)	${{\boldsymbol{d}}}_{(11\bar{{\bf{2}}}0)}$(Å)	${{\boldsymbol{d}}}_{(\bar{{\bf{2}}}200)}$(Å)	*ε*_*xx*_ (%)	*ε*_*yy*_ (%)
[11$\bar{2}$0] (*x*)	1.5921	—	−2.197	—	1.6087	—	−1.178	—
[$\bar{1}$100] (*y*)	—	1.3788	—	−2.197	—	1.3827	—	−1.921

**Figure 4:**
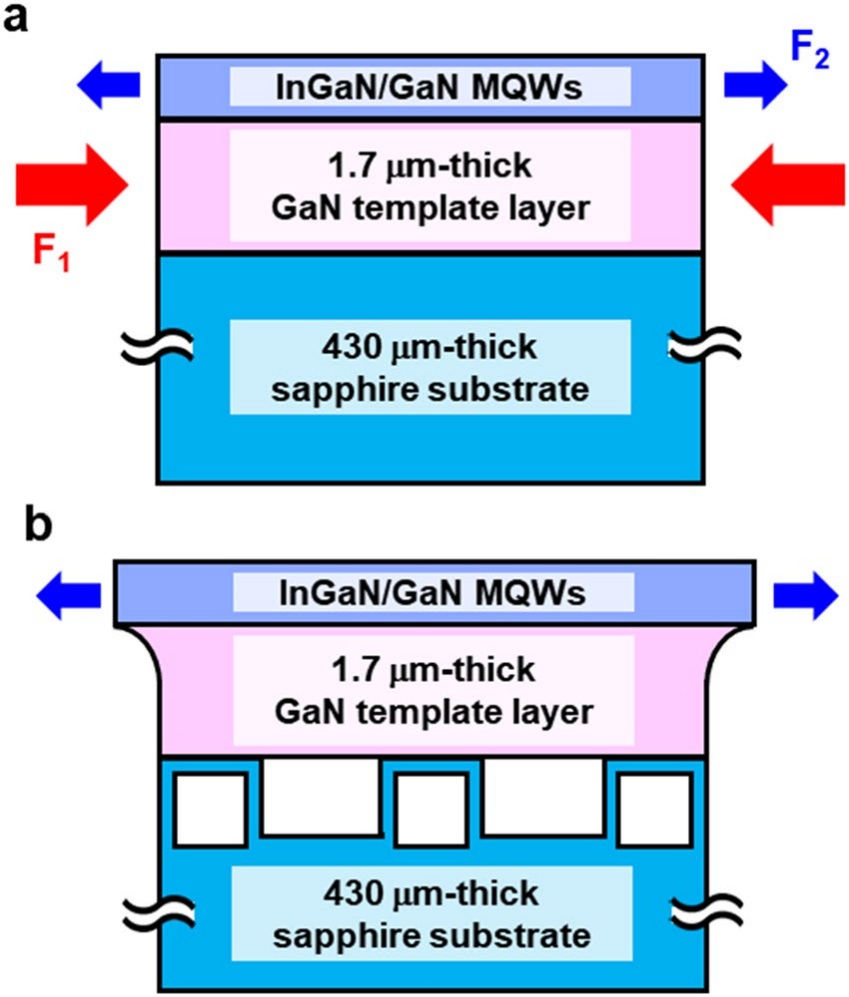
Investigation on validity of a structural model of InGaN/GaN MQWs on GaN/SCES template. Structural model for (**a**) reference sample and (**b**) InGaN/GaN MQWs on the GaN/SCES template and forces in the epitaxial layers.

From the RSM results, we propose a structural model for the InGaN/GaN MQWs on the GaN/SCES template as shown in Fig. 4b. For the reference sample, the 430 μm-thick sapphire substrate was in complete contact with the GaN template layer. Due to the difference in thermal expansion coefficient between GaN and sapphire, GaN was under severe compressive force so that local deformation of the template layer was not allowed, as shown in Fig. 4a. On the other hand, the cavity-incorporated nano-membranes in SCES can reduce the influence of sapphire substrate on the GaN template layer. The InGaN layer with a larger lattice parameter compared to GaN can locally expand the GaN template layer near the interface with InGaN/GaN MQWs, as shown in Fig. 4b. The InGaN/GaN MQWs were pseudomorphically grown on the deformed region of the GaN template layer. We investigated validity of the structural model by comparing the compressive force (*F*_1_) induced by thermal strain in GaN template with the tensile force (*F*_2_) due to lattice mismatched strain between InGaN and GaN in the InGaN well (See Supplementary Information). The compressive force was about 70 times larger than the tensile force, implying that severe compressive force induced by thermal strain should suppress the local deformation of the GaN template layer. On the other hand, the influence of the sapphire substrate was reduced for the InGaN/GaN MQWs on the SCES, resulting in the deformation of the GaN template, as shown in Fig. 4b.

However, the extents of elastic deformation of the GaN template layer were anisotropic due to the anisotropic alignment of cavity-incorporated nano-membranes. Along the *x*-direction, the continuous epitaxial layers were periodically touched with the 2 μm width stripe-shaped nano-membranes at the pitch of 4 μm, leading to effective local deformation of the GaN template layer. On the other hand, the membranes were in full contact with the epitaxial layer along the *y*-direction so that extent of deformation of GaN layer was relatively limited, resulting in the significant anisotropic in-plane strain states in InGaN wells on the GaN/SCES template.

Optical properties of the samples were analyzed by a micro-PL system with a 325 nm He-Cd laser at room temperature. About four times higher PL intensity was observed from the InGaN/GaN MQWs on GaN/SCES template compared to the reference sample due to the improved crystalline quality^34^, the partial strain relaxation in the InGaN wells, and resultant reduced quantum confinement Stark effect (QCSE)^30^. From the enhancement of PL intensity, increased electroluminescence signals can be expected in actual LEDs fabricated with the SCES. The reduction of QCSE also induced the blue-shift of peak position of 11 meV compared to the reference sample (See Supplementary Information)^30^.

**Figure 5:**
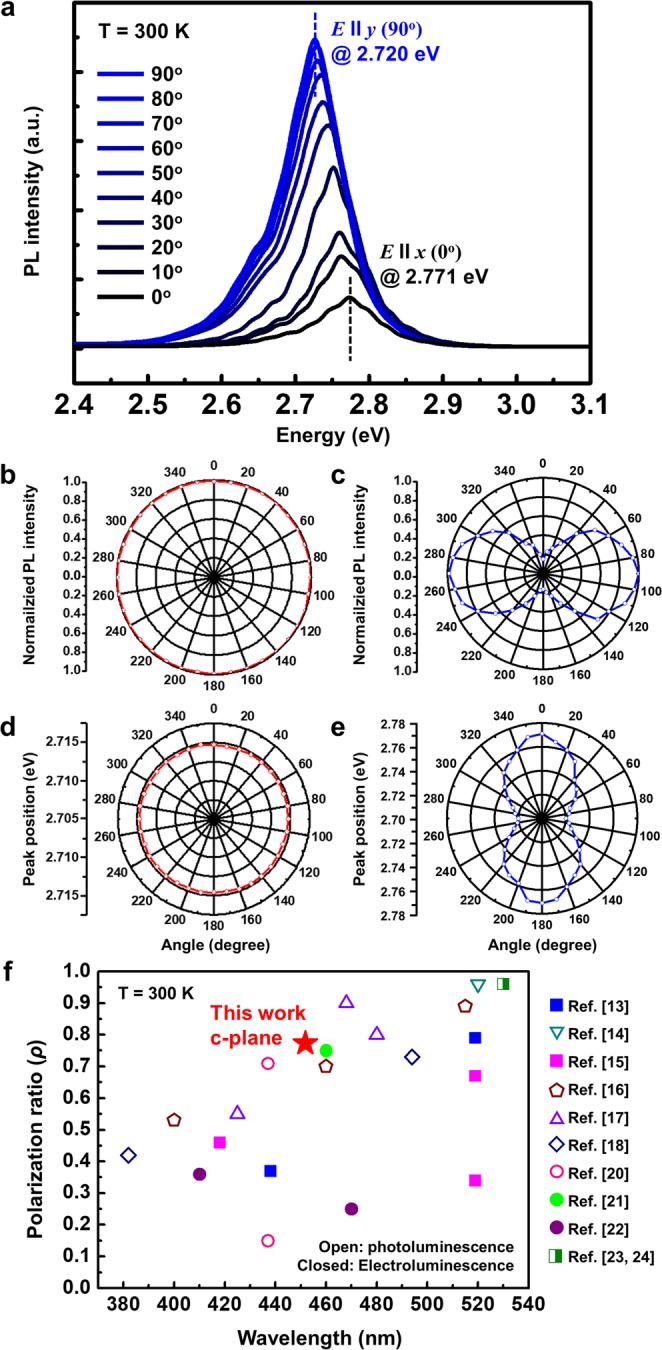
Investigation on polarized optical behaviors of InGaN/GaN MQWs on GaN/SCES template and reference sample. (**a**) PL spectra from InGaN/GaN MQWs on GaN/SCES template with polarizer angles from 0° to 90°_._ Normalized PL intensities from (**b**) reference sample and (**c**) InGaN/GaN MQWs on GaN/SCES template, and peak positions of (**d**) reference sample and (**e**) InGaN/GaN MQWs on GaN/SCES template as a function of rotational polarizer angle. (**f**) Polarization ratios from various InGaN/GaN MQWs structures.

From the results of RSM measurements, modification of valence band structures and resultant polarized light emission from InGaN/GaN MQWs grown on the GaN/SCES template were also expected^34,39^. To measure the polarized PL, a rotating linear polarizer was placed between the samples and a detector. The polarizer angles of 0° and 90° corresponded to the directions of electric-field vector *E* parallel to *x* (*E***||***x*) and *E* parallel to *y* (*E***||***y*), respectively. PL spectra from InGaN/GaN MQWs on the GaN/SCES template measured over an angular range from 0° to 90° were shown in Fig. 5a. InGaN/GaN MQWs on the GaN/SCES template showed typical polarized optical behaviors from III-nitride semiconductors such as gradual enhancement of the PL intensities and corresponding red-shift of the peak wavelength from *E***||***x* to *E***||***y* directions. Figure 5b,d show polarizer angle-dependent normalized PL intensities and peak positions of the reference sample, respectively. Those of InGaN/GaN MQWs on the GaN/SCES template are shown in Fig. 5c,e, respectively. As expected, any significant changes of PL intensities and peak shifts were not observed from the InGaN/GaN MQWs of the reference sample due to its in-plane symmetry and resultant isotropic in-plane strain. On the other hand, the polarized optical behaviors of InGaN/GaN MQWs on the GaN/SCES template were clearly shown in Fig. 5c,e. The polarization ratio (*ρ*) [defined as (*I*_*max*_ *−* *I*_*min*_)/(*I*_*max*_ + *I*_*min*_)]^20^ was found to be as high as 0.74, where *I*_*max*_ and *I*_*min*_ were maximum and minimum integrated PL intensities, respectively. The polarization ratio obtained in this study was compared with the values previously reported in the literatures^13–18,20–24^. Polarization ratios were plotted for various InGaN/GaN MQWs as shown in Fig. 5f. Most of previous studies utilized the non- and semi-polar InGaN/GaN MQWs grown on bulk GaN substrates or hetero-epitaxial structures. Studies on c-plane InGaN/GaN MQWs on c-plane sapphire substrate adopted the complex post-processes which included the reduction of active region area, or losses of light. On the other hand, anisotropic in-plane strain states in c-plane InGaN wells on c-plane sapphire substrate were induced by SCES, leading to the realization of polarized light emission (marked by star), in this study. Moreover, we believe that extent of anisotropy in strain in InGaN/GaN MQWs should be enlarged by controlling the shapes, dimension, and alignment of the nano-membrane structures, which may lead to improvement in the polarization ratio.

**Figure 6:**
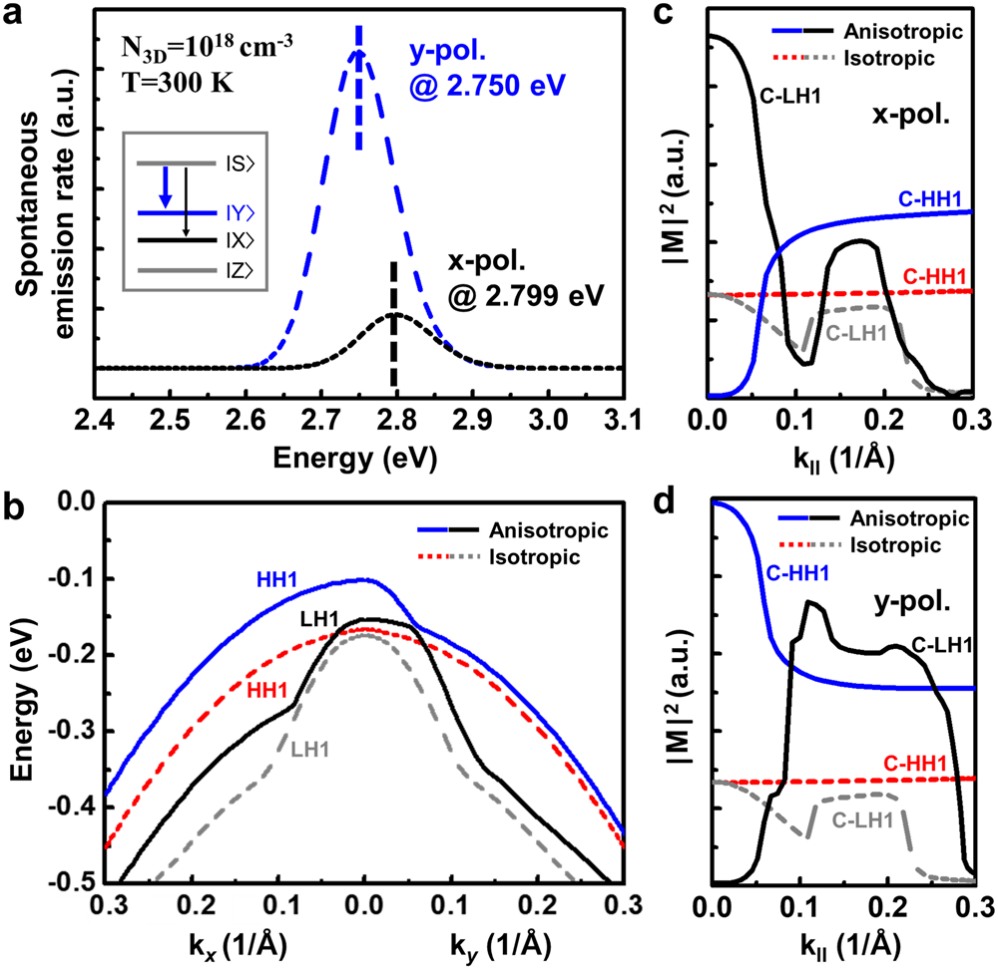
Theoretical calculations based on *k*∙*p* perturbation theory under anisotropic and isotropic strain states. (**a**) Calculated *x*- and *y*- polarized spontaneous emission rates under the anisotropic in-plane strain. (**b**) Valence band structures under anisotropic and isotropic strain states. (**c**) *x*-polarized and (**d**) *y*-polarized optical matrix elements under anisotropic and isotropic strain states.

To find out the fundamental origin of the polarized light emission, theoretical calculations based on *k∙p* perturbation theory were conducted by solving 6 × 6 Hamiltonian matrix^39^. Figure 6a shows *x*- and *y*-polarized spontaneous emission rates of anisotropically-strained InGaN/GaN MQWs. We calculated the spontaneous emission rates by combining valence band structures and optical matrix elements (${\mid}M{\mid}$^2^), which determine recombination probabilities of electrons and holes from each conduction and valence subband. The details of theoretical calculations were as described elsewhere^40^. The strain states in InGaN wells on the GaN/SCES template measured from the RSM measurements (−1.178%, −1.921%), indium compositions (19.5%), and thicknesses of InGaN well (2.5 nm) and GaN barrier (7.5 nm) were substituted into the theoretical calculations. Material parameters and band gaps of GaN and InN were taken from the literatures^41,42^. Deformation potentials (*a*, *D*) and valence band effective-mass parameters (*A*) used in the calculations were summarized in Table 2. Data are from ref.^41^. As shown in the Fig. 6a, the calculated values of polarization ratio of 0.71 and peak wavelength shifts of 49 meV were in good agreement with the experimental results of 0.74 and 51 meV, respectively. This good agreement indicates that the linearly polarized light emission from the InGaN/GaN MQWs on GaN/SCES template was attributed to the modification of valence band structures induced by the anisotropic in-plane strain in the MQWs. The conduction bands of wurtzite III-nitride semiconductors are composed of symmetric *s* orbitals with $|S\rangle$-like wave function. The valence bands of unstrained or isotropically strained III-nitride semiconductors are comprised of 2*p* orbitals with $|X\pm iY\rangle$-like wave functions for heavy hole (HH) and light hole (LH) bands, and $|Z\rangle$-like wave function for spin-orbit crystal-field split-off hole band. Anisotropic in-plane strain in c-plane III-nitride semiconductors breaks the degeneracy of topmost valence subbands, leading to the polarized optical light emission, as shown in the inset of Fig. 6a^20,34^. The calculated valence band structures of anisotropically and isotropically strained InGaN/GaN MQWs were shown in Fig. 6b. Under the isotropic strain states, energy level differences at Brillouin zone center (k = 0) between the topmost HH1 and LH1 subbands were 8.4 meV, which results from the spin-orbit splitting energy^39^. On the other hand, the anisotropic strain split and enlarged those between HH1 and LH1 subbands to 50.9 meV, which are comparable with peak wavelength shifts. The isotropic valence band structures under isotropic strain also anisotropically changed under anisotropic strain states. Figure 6c,d show *x*-polarized and *y*-polarized optical matrix elements, respectively. The notation k_**||**_ indicates that the optical matrix elements were averaged in k_*x*_–k_*y*_ plane. The *x*-polarized and *y*-polarized optical matrix elements under the isotropic strain were same, whereas those under the anisotropic strain exhibited different behaviors near the Brillouin zone center. The *x*-polarized light emission is dominant from the transition between conduction band and LH1 (C-LH1) rather than C-HH1, whereas *y*-polarized light emission is larger from C-HH1 than C-LH1 as shown in Fig. 6c,d, respectively. As a result, relatively higher hole concentrations in HH1 than LH1 induced larger *y*-polarized light emission than *x*-polarized one.

**Table 2:** Deformation potentials and valence band effective-mass parameters of GaN and InN^41^.

Deformation potentials (eV)	GaN	InN
*a*_*c*_ (conduction band)	−4.60	−1.40
*D*_1_	−1.70	−1.76
*D*_2_	6.30	3.43
*D*_3_	8.00	5.19
*D*_4_	−4.00	−2.60
*D*_5_	−4.00	−2.33
Valence band effective-mass parameters		
*A*_1_	−6.40	−9.09
*A*_2_	−0.50	−0.63
*A*_3_	5.90	8.46
*A*_4_	−2.95	−4.23
*A*_5_	−2.56	−4.36
*A*_6_	−3.07	−6.35

## Conclusions

In summary, five-period c-plane InGaN/GaN MQWs were grown on GaN/SCES template to realize polarized light emission from the MQWs. We reported the anisotropic in-plane strain states in the InGaN wells by using the anisotropically arranged cavity-incorporated nano-membrane structures. Consequently, linearly polarized PL emission from MQWs was achieved and the polarization ratio was as high as 0.74. The results of in-plane polarization anisotropy were explained in terms of the strain-induced valence band modification by comparing the experimental results with calculated values based on *k∙p* perturbation theory. This study gives opportunities to realize highly efficient LEDs with high polarization ratio on c-plane sapphire substrates by strain-induced modification of valence band structures of III-nitride semiconductors.

## Method

### Fabrication of SCES

Stripe-shaped photoresist (PR) was defined on a 2 inch sapphire substrate along the [11$\bar{2}$0] direction of sapphire substrate, considering the anisotropic growth behavior of GaN to be grown on the SCES. A 120 nm-thick amorphous alumina layer were deposited on the PR-patterned sapphire substrate by atomic layer deposition. The edge area of the sample was scraped and PR inside the amorphous alumina layer was removed by acetone, resulting in arranged cavity-incorporated amorphous alumina membranes. After PR removal using acetone, the sample was subsequently annealed at 800 °C and 1100 °C for 1 and 2 hours, respectively. During the thermal annealing, the amorphous alumina layer was crystallized into single crystalline **α**-phase Al_2_O_3_ (same with sapphire substrate) via γ-phase Al_2_O_3_ as described in our previous study^43^. Fabrication procedures of the SCES were described in detail elsewhere^34^.

### Epitaxial growth

The fabricated SCES and a planar sapphire substrate as a reference sample were loaded into the MOCVD chamber. 1.7 μm-thick continuous GaN template layer was grown on the SCES and planar sapphire substrate after 90 min growth. The growth of GaN template layer was described in detail elsewhere^34^. The InGaN quantum well and GaN barrier layers were grown at 750 °C and 800 °C, respectively, under pressure of 30 kPa. Trimethylgallium, trimethylindium, and ammonia were used as precursors for Ga, In, and N, respectively. Hydrogen and nitrogen were used as carrier gases during the growth of GaN and InGaN, respectively.

### Chracterization

Bird’s eye view images of SCES and epitaxial layers were obtained by a Hitach S-4800 FE-SEM. Cross-section STEM and reconstructed high-resolution TEM images obtained by JEOL JEM-2100F. High-resolution XRD and RSM measurement were conducted using a PANalytical X’pert Pro triple-axis diffractometer equipped with a 4-bounce Ge (220) hybrid monochromator.

### Theoretical calculations

Valence band structures and optical matrix elements, and spontaneous emission rates were calculated by solving 6 × 6 Hamiltonian matrix using self-designed Fortran programs.

## Supplementary information

Supplementary information

## Data Availability

All data generated or analyzed during this study are included in this published article (and its Supplementary Information Files).
