# Supplementary material for: Highly polarized photoluminescence from c-plane InGaN/GaN multiple quantum wells on stripe-shaped cavity-engineered sapphire substrate
Source: Sci Rep. 2019 Jun 4;9:8282. doi: 10.1038/s41598-019-44519-2 (PMC6547675; doi:10.1038/s41598-019-44519-2)
Supplement: Supplementary file 1 — Supplementary information [file 41598_2019_44519_MOESM1_ESM.pdf]

Supplementary information

Highly polarized photoluminescence from c-plane InGaN/GaN multiple quantum wells on stripe-shaped cavity-engineered sapphire substrate

Jongmyeong Kim,[1] Seungmin Lee,[1] Jehong Oh,[1] Jungel Ryu,[1] Yongjo Park,[1] Seoung-Hwan Park,[2] and Euijoon Yoon[1,3,4]*

[1] *Department of Materials Science and Engineering, Seoul National University, Seoul 08826, Korea*

[2]*Department of Electronics Engineering, Catholic University of Daegu, Gyeongbuk 38430, Korea*

[3]*Research Institute of Advanced Materials, Seoul National University, Seoul 08826, Korea*

[4] *Inter-university Semiconductor Research Center, Seoul National University, Seoul 08826, Korea*

**E-mail: eyoon@snu.ac.kr*

Contents

- Calculations of interplanar spacing and corresponding strains
- Investigation on validity of a structural model
- Analysis on optical properties of InGaN/GaN MQWs of samples

Calculations of interplanar spacing and corresponding strains from high-resolution X-ray reciprocal space mapping (RSM) measurements

The interplanar spacing (*d*) and corresponding strains in InGaN wells along the *x* and *y* directions (ε_{xx} and ε_{yy}) were calculated from the reciprocal lattice points (RLPs) using the equations as follows[1]:

$$d_{(11\bar{2}0)} = \frac{1}{\sqrt{(Q_x^{(11\bar{2}4)} - Q_x^{(0004)})^2 + (Q_z^{(11\bar{2}4)} - Q_z^{(0004)})^2}}, \tag{S1}$$

$$d_{(\bar{2}200)} = \frac{1}{\sqrt{(Q_x^{(\bar{2}204)} - Q_x^{(0004)})^2 + (Q_z^{(\bar{2}204)} - Q_z^{(0004)})^2}}, \tag{S2}$$

$$\varepsilon_{xx} = \frac{d_{(11\bar{2}0),well} - d_{(11\bar{2}0),bulk}}{d_{(11\bar{2}0),bulk}}, \tag{S3}$$

$$\varepsilon_{yy} = \frac{d_{(\bar{2}200),well} - d_{(\bar{2}200),bulk}}{d_{(\bar{2}200),bulk}}, \tag{S4}$$

where λ and (Q_x, Q_z) are wavelength of X-ray and coordinates of RLPs, respectively. Each interplanar spacing of the bulk InGaN was calculated by using the lattice parameters of InGaN obtained from the Vegard's law. Material parameters of GaN and InN were taken from the literature[2].

Investigation on validity of a structural model for reference sample and InGaN/GaN MQWs on GaN/SCES template

Validity of the structural model for InGaN/GaN MQWs were investigated by comparing compressive force (F_1) induced by thermal strain in GaN template with tensile force (F_2) due to lattice mismatch between InGaN and GaN in the the InGaN wells as shown in Fig. S1.

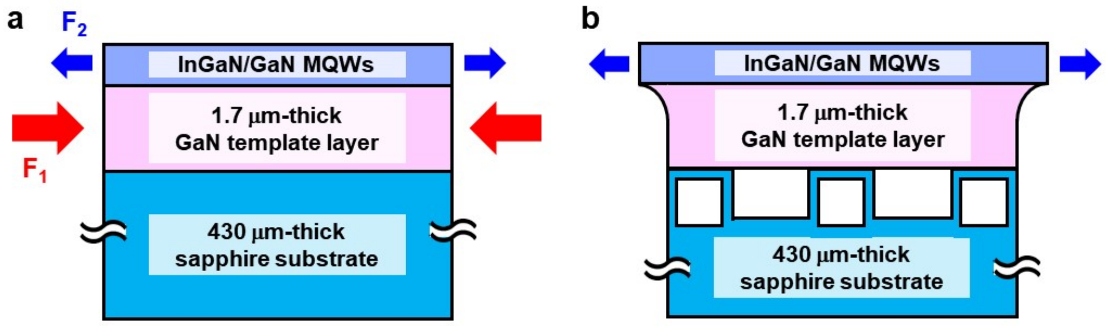

Fig. S1. Structural model for InGaN/GaN MQWs and forces in the epitaxial layers.

Compressive force (F_1) in GaN template layer on sapphire substrate was calculated as follows;

$$\varepsilon_T = (\alpha_{GaN} - \alpha_{Sapphire}) \times \Delta T \,, \tag{S5}$$

$$\sigma_T = M_{GaN} \times \varepsilon_T \,, \tag{S6}$$

$$F_1 = \sigma_T \times t_{GaN} \times W \,, \tag{S7}$$

where ε_T, α, ΔT, σ_T, M_{GaN}, t_{GaN}, and W are thermal strain, coefficient of thermal expansion[1], difference between growth and room temperatures, thermal stress, biaxial modulus of GaN[3], thickness of GaN template layer, and width of the sample, respectively. Here, the sample width was 1 m for the convenience of calculations.

Tensile force (F_2) in InGaN well was calculated as follows;

$$\varepsilon_L = \frac{a_{bulk}^{GaN} - a_{bulk}^{InGaN}}{a_{bulk}^{InGaN}}, \tag{S5}$$

$$\sigma_L = M_{InGaN} \times \varepsilon_L, \tag{S6}$$

$$F_2 = \sigma_L \times t_{InGaN} \times W, \tag{S7}$$

where ε_L, a, σ_L, M_{InGaN}, t_{InGaN}, and W are lattice mismatch strain, in-plane lattice constant[1], lattice mismatch stress, biaxial modulus of InGaN[3,4], thickness of InGaN well, and width of the sample, respectively. Here, the sample width was 1 m for the convenience of calculations.

Compressive force (-1496.35 N) was about 70 times larger than tensile force (20.45 N), implying that severe compressive force induced by thermal strain should suppress the local deformation of the GaN template layer. On the other hand, the influence of the sapphire substrate was reduced for the InGaN/GaN MQWs on the SCES, resulting in the deformation of the GaN template, as shown in Fig. S1b.

Analysis on optical properties of InGaN/GaN MQWs grown on GaN/SCES template and reference sample

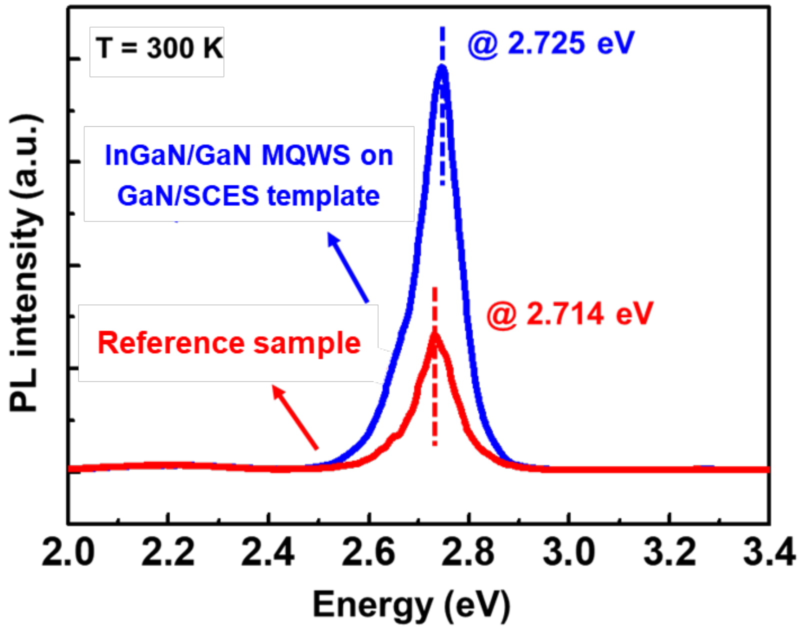

Fig. S2. PL spectra from reference sample and InGaN/GaN MQWs on GaN/SCES template.

Optical properties of the samples were analyzed by photoluminescence (PL) measurements. PL spectra from the reference sample and InGaN/GaN MQWs on the GaN/SCES template were shown in Fig. S2. The integrated PL intensity from the InGaN/GaN MQWs GaN/SCES tempalte was about four times higher than that of the reference sample. We believe the enhancement of PL intensities were attributed to the improved crystalline quality of GaN as reported in our earlier study[5] as well as reduced quantum confine Stark effect (QCSE) due to the partial strain relaxation in InGaN wells[6]. Blue-shift of peak positon of 11 meV in InGaN/GaN MQWs on the GaN/SCES template compared to the reference sample were also due to the reduced QCSE[6].

Reference

1 Kidd, P. XRD of Galluim Nitride and Related Compounds: Strain, Composition, and Layer Thickness (Panalytical, Almelo, 2009).

2 Park, S. H., Ahn, D. & Shuang, S. L. Electronic and optical properties of a- and m-plane wurtzite InGaN-GaN quantum wells. *IEEE J. Quant. Electron.* **43**, 1175 (2007).

3 Hearne, *S. et al.* Stress evolution during metalorganic chemical vapor deposition of GaN. *Appl. Phys. Lett.* **74**, 356 (1999).

4 Kim, K., Lambrecht, W. R. L., & Segall, B. Elastic constants and related properties of tetrahedrally bonded BN, AlN, GaN, and InN. *Phys. Rev. B* **56**, 7018 (1997).

5 Kim, J. *et al.* Linearly polarized photoluminescence of anisotropically strained c-plane GaN layers on stripe-shaped cavity-engineered sapphire substrate. *Appl. Phys. Lett.* **112**, 212102 (2018).

6 Wang, Q., Bai, J., Gong, Y. P. & Wang, T. Influence of strain relaxation on the optical properties of InGaN/GaN multiple quantum well nanorods. *J. Phys. D: Appl. Phys.* **44**, 395102 (2011).
